# T-DM1 efficacy in trastuzumab-pertuzumab pre-treated HER2 positive metastatic breast cancer patients: a meta-analysis

**DOI:** 10.1186/s12885-022-09556-7

**Published:** 2022-06-07

**Authors:** Claudia Omarini, Federico Piacentini, Isabella Sperduti, Krisida Cerma, Monica Barbolini, Fabio Canino, Cecilia Nasso, Christel Isca, Federica Caggia, Massimo Dominici, Luca Moscetti

**Affiliations:** 1 Division of Medical Oncology, University Hospital of Modena, Via del Pozzo 71, 41122 Modena, Italy; 2 Department of Medical and Surgical Sciences for Children & Adults, University Hospital of Modena, Modena, Italy; 3 Department of Bio-Statistics, RCCS Regina Elena National Cancer Institute, 00144 Rome, Italy

**Keywords:** HER2 positive, T-DM1, Pertuzumab, Trastuzumab emtansine, Metastatic breast cancer

## Abstract

**Background:**

Current guidelines consider T-DM1 the standard 2^nd^ line therapy for HER2 positive metastatic breast cancer (MBC) patients following trastuzumab (T) + pertuzumab (P) and taxane 1^st^ line treatment. Despite this, there are no prospective studies supporting this sequence.

**Methods:**

We performed a meta-analysis using real world data to determine the efficacy of T-DM1 after 1^st^ line TP in HER2 positive MBC patients. We used a random-effect model to find differences in the rate of 1-year progression free survival (PFS) between TP pre-treated population and the EMILIA phase III pivotal trial.

**Results:**

Seven studies were eligible. The meta-analysis showed a combined 1-year PFS risk difference for T-DM1 efficacy after TP in 2^nd^ or more lines of -0.122, with lower and upper limits of -0.253 and 0.010, respectively (*p* = 0.07), with low heterogeneity among studies (I^2^ 0.01%, *p* = 0.836). Considering the four studies on T-DM1 in 2^nd^ line setting, 1-year PFS risk was -0.034 (95% CI -0.207 – 0,139; *p* = 0.701) (I^2^ 0.01%, *p* = 0.91).

**Conclusion:**

Overall, the efficacy of T-DM1 after TP seems to be similar to that previously reported in the EMILIA trial. In the second line setting, data are not mature enough to confirm T-DM1 efficacy in TP pre-treated population.

**Supplementary Information:**

The online version contains supplementary material available at 10.1186/s12885-022-09556-7.

## Background

Amplification of the HER2 gene is present in 15–20% of tumours in patients with breast cancer. In the past, a diagnosis with HER2-positive breast cancer was associated with a poor prognosis. Advances in drug therapies in recent years have improved the treatment options for HER2-positive breast cancer (BC) as well as the outlook of these patients. Trastuzumab (T), a humanised IgG1 monoclonal antibody that targets the extracellular domain of the HER2 protein, administered alone or in combination with chemotherapy, improves survival outcomes of HER2-positive metastatic breast cancer [1].

T-DM1 is an antibody–drug conjugate composed of trastuzumab plus a cytotoxic drug (emtansine) that directly targets the HER2-expressing cells [1]. Current international guidelines consider the sequence of first line (1^st^) double blockade with T and pertuzumab (P) plus taxane followed by T-DM1 as the optimal management of HER2 positive metastatic breast cancer (MBC) patients [2]. From publication of the phase III EMILIA trial results, T-DM1 has been considered the preferred second (2^nd^) line choice due to the gain in survival outcomes compared to the control arm of capecitabine and lapatinib [3]. Data from the Phase III TH3RESA study confirmed T-DM1 efficacy in heavily pretreated patients too [4]. However, there were no TP pre-treated patients in either the EMILIA or TH3RESA study populations. To date, there are no published randomized trials addressing the efficacy of T-DM1 in TP pre-treated HER2 positive MBC. Available data are mainly from real-word experiences that have investigated this sequence with controversial results [5–14].

The present meta-analysis aimed to investigate the efficacy of T-DM1 after the combination of TP in HER2 positive MBC patients. In particular, we focused on the activity of T-DM1 in 2^nd^ line setting following TP and taxane 1^st^ line treatment.

## Methods

The search strategy was designed by CO and LM and approved by all the other authors. The systematic literature research was conducted by KC, CI, MB, CN and FaC. The terms used for the research were: “breast cancer” and “T-DM1”. Boolean operators were used to connect specific search keywords for each database and other free text terms. The search was conducted using electronic databases: PubMed, EMBASE (from 1946), Cochrane Library (2018) and Web of Science (from 1900). We collected all studies, already published as full-text articles regarding the efficacy of T-DM1 in TP pre-treated HER2 positive MBC patients.. In order to find any additional eligible trials, the references reported in the selected papers were also checked. No language restriction or restriction in terms of year of publication were applied. The final date for running database searches was December 31^st^ 2020. The flow of eligible articles followed the Preferred Reporting Items for Systematic Reviews and Meta-Analyses (PRISMA) statement. An objective assessment of the methodologic quality of the retrospective studies was conducted according to the checklists of the Strengthening the Reporting of Observational Studies in Epidemiology (STROBE) statement (supplementary material Table 1). Data collected included: first author name, name of the trial if available, year of publication, sample size of TP pre-treated patients and the rate of patients still on T-DM1 after 1-year of treatment. T-DM1 efficacy was evaluated by 1-year Progression free survival (PFS) calculated from the treatment start date to the date of T-DM1 discontinuation due to disease progression. When not directly available, the 1-year PFS rate was calculated by the authors (CO and LM) using the Kaplan–Meier method. Articles with no available Kaplan–Meier curves for PFS in the subgroup of TP pre-treated patients were excluded from the final analysis. The 1-year PFS rate in TP pretreated patients was compared to 1-year PFS rate reported in EMILIA. Finally, a meta-analysis including studies on T-DM1 in 2^nd^ line setting only were also performed.

**Table 1:** Clinical trials included trastuzumab pertuzumab pre-treated metastatic breast cancer population. The second part of the table reported the data from the two phases III trials EMILIA and TH3RESA included in the analysis as comparators

**First author name (Trial)**	**Year**	**Previous treatment**	**T-DM1 line**	**PFS** months	**95%CI**	**Patients N**	**1-year PFS** % patients
Dzimitrowicz et al. [5]	2016	TP + CHT or ET	2nd and further	4	2,7—5,1	78	*NA*
Conte et al. [8]	2019	TP TXT	2nd-line	6,3	4,8—7,7	77	28
Urruticoechea et al. [14] (CLEOPATRA) (PHEREXA)	2017	TP TXT	2nd and further	7,1	0—44	32	*NA*
	2017	TPC	2nd and further	4,2	0—22	43	*NA*
Fabi et al. [9]	2017	TP TXT	2nd-line	5	4,3—5,7	34	48
Noda-Narita et al. [10]	2019	TP TXT	2nd and further	2,8	1,7—4,8	18	12
Vici et al. [11]	2017	TP TXT	2nd and further	4	2—7	47	13,2
Lupichuk et al. [12]	2019	TP TXT	2nd and further	5,5		55	22
Battisti et al. [13]	2020	TP TXT	2nd and further	8,7	6,6—11,3	37	*NA*
Del prete et al. []	2020	TP TXT	2nd	10,5	8,6—12,7	135	47
Huober et al. (PERNETTA) [6]	2018	TP	2nd	7,1	4,3—11,9	59	30
	2018	TP TXT	2nd	5,3	4—10,3	42	32
**Comparator Phase III trial**							
Krop et al. (EMILIA) [4]	2012	T TXT	2nd	9,6	0,55 – 0,77	495	40

*T* Trastuzumab, *P* Pertuzumab, *TXT* taxane, *C* Capecitabine, *CHT* Chemotherapy regimen, *PFS* progression free survival

### Statistical analysis

One-year PFS was calculated from the start date of T-DM1. For each study, the standard error (SE) of the 1-year mortality rate was calculated according to the formula: SE = √ (a x (1-a) / √ (n) where a = 1-year rate and n = sample size [15]. The difference in 1-year PFS between the real world study population and the pivotal trial population was calculated for each study. The standard error (SE) of the difference was calculated as SE (diff) = √ ((SE1)^2 + (SE2)^2) where SE1 is the SE of the 1-year PFS rate of the real world study population and SE2 is the SE of the 1-year PFS rate of the pivotal trial population. The Q statistics were used to test for heterogeneity between the studies included in the meta-analyses. The proportion of total variability attributed to between-study heterogeneity was assessed with the I^2^ statistic, a confirmatory test for heterogeneity, with I^2^ less than 25%, 25% to 50%, and greater than 50% representing low, moderate, and high degrees of heterogeneity, respectively. Results are graphically displayed as forest plots, with rate of difference < 0 indicating that the efficacy of T-DM1 in real word data was similar to that reported in the EMILIA pivotal trial. Statistical significance was set at p < 0.05. Independently of the degree of heterogeneity observed, a random-effect model was identified as the most appropriate approach. Results were also derived from a fixed-effect model. A funnel plot was used for publication bias assessment. Calculations were performed using the Comprehensive Meta-Analysis Software, version v. 2.0 (CMA, Biostat, Englewood, NJ, USA) [16].

## Results

**Fig. 1:**
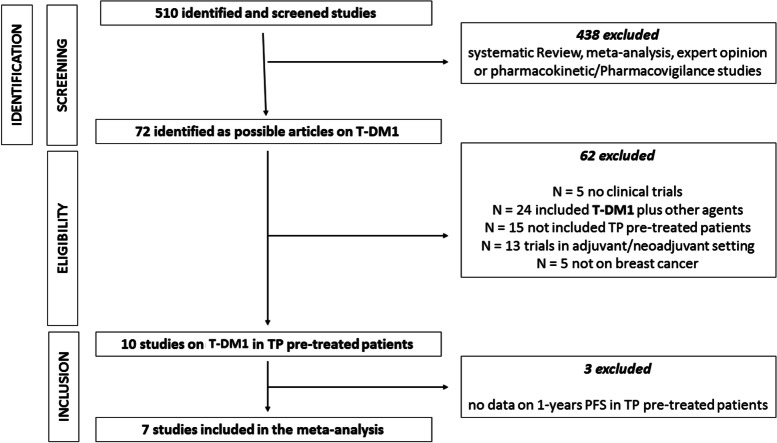
PRISMA flow chart summarizing the process to identify the eligible studies

According to our research strategy, we identified 510 publications. Based on the information found in their title and/or abstract, 438 were classified as systematic review, meta-analysis, expert opinion or pharmacokinetic/pharmacovigilance studies. Among the remaining 72 articles, 67 were clinical trials. Fifty-seven of them did not meet the inclusion criteria (24 were clinical trials evaluating the efficacy of T-DM1 plus other antineoplastic drugs, 15 studies did not include TP pre-treated patients, 13 trials were conducted in adjuvant/neoadjuvant setting and 5 were not on BC) (Fig. 1). Among the remaining 10 trials, 3 were excluded because the 1-year PFS rate for TP pre-treated population was not available (Table 1). Of note, the PERNETTA trial included two different TP pre-treated populations, with and without taxane, that have been considered as two different study populations in the meta-analysis [6]. Finally, eight studies were suitable for our meta-analysis. All but one (PERNETTA trial) were retrospective data and in three of them data were from sub-group population analysis. In particular, the PERNETTA trial was a non-comparative randomized open label phase II trial of PT with or without chemotherapy, both followed by T-DM1 in case of progression. Study characteristics were reported in Table 1. Considering T-DM1 in 2^nd^ line setting only, 5 trials investigated this issue.

### Comparison between T-DM1 efficacy in TP pre-treated patients and EMILIA and TH3RESA trial populations

**Fig. 2:**
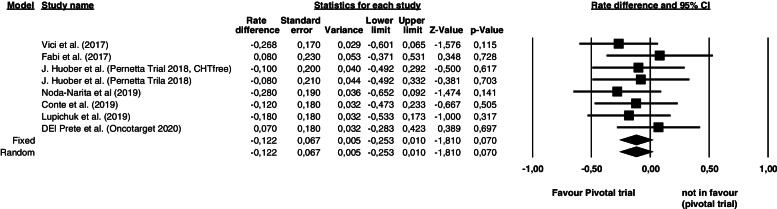
Comparisons of 1-year Progression Free Survival (PFS) of T-DM1 between trastuzumab pertuzumab pre-treated population and trastuzumab pre-treated population (EMILIA and TH3RESA trials). The left part of the figure shows the studies included in the analysis with their corresponding rate difference, standard error, lower and upper limits, z-value and p-value while the right part of the figure shows a forest plot of the data. The square represents the risk difference for each study, the horizontal lines represent the values within the 95% confidence interval 8CI) of the underlying effects. The vertical line represents a risk difference of 0

Overall, the meta-analysis showed a combined 1-year PFS risk difference for T-DM1 efficacy after TP in 2^nd^ or further lines of -0.122, with lower and upper confident interval limits of -0.253 and 0.010, respectively (*p* = 0.07), with low heterogeneity among studies (I^2^ 0.01%, *p* = 0.836) (Fig. 2). Regardless of T-DM1 treatment lines, in the TP pre-treated population, the efficacy rate of T-DM1 does not seem to be any lower than those reported in the randomized Phase III EMILIA trial with a *p* value = 0.07.

### Comparison between 2^nd^ line T-DM1 efficacy in TP pre-treated patients and EMILIA trials

**Fig. 3:**
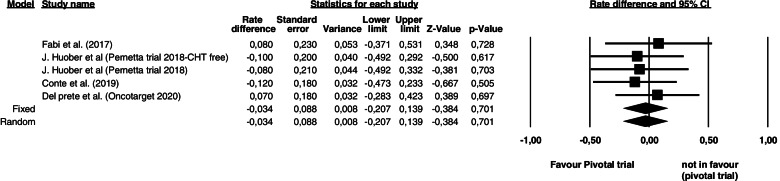
Comparisons of 1-year Progression Free Survival (PFS) of T-DM1 in 2nd line between trastuzumab pertuzumab pre-treated population and EMILIA trial population. The left part of the figure shows the studies included in the analysis with their corresponding rate difference, standard error, lower and upper limits, z-value and p-value while the right part of the figure show a forest plot of the data. The square represents the risk difference for each study, the horizontal lines represent the values within the 95% confidence interval 8CI) of the underlying effects. The vertical line represents a risk difference of 0

**Fig. 4:**
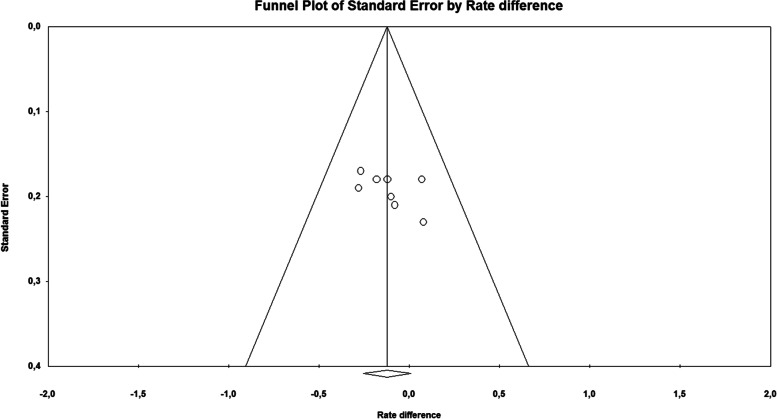
Funnel plot of Standard Error by rate difference showing that all the studies fall symmetrically, suggesting a lack of significant publication bias. Circles indicated individual studies, dotted lines indicated pooled risk differences, and black lines were for estimating symmetry

Considering the five studies on T-DM1 in 2^nd^ line setting, the 1-year PFS risk was -0.034 (95% CI -0.207 – 0,139; *p* = 0.701), with low heterogeneity among studies (I^2^ 0.01%, *p* = 0.91) (Fig. 3). Our results show that the available data are not mature enough to confirm the efficacy of T-DM1 as 2^nd^ line treatment in a TP pre-treated population (*p* = 0.91) (Fig 4).

## Discussion

The role of T-DM1 after dual anti-HER2 therapy blockage in the treatment of HER2 + MBC patients is currently under discussion. The pivotal study is the Phase III EMILIA trial, which investigated 2^nd^ line T-DM1 activity in patients pre-treated with trastuzumab and taxane, a regimen that is now considered suboptimal [3]. The reported survival outcomes in the T-DM1 arm were: objective response rate (ORR) of 43.6%, median PFS of 9.6 months (hazard ratio 0.65; 95% CI 0.55 to 0.77; *P* < 0.001) and median OS of 30.9 months (hazard ratio 0.68; 95% CI, 0.55 to 0.85; *P* < 0.001) [3]. Unfortunately, no TP pre-treated patients were included in the EMILIA study population. For this reason, T-DM1 efficacy after the dual block containing P is still unknown. Discordant conclusions emerged from a series of retrospective real world studies published [5–14]. In particular, T-DM1 efficacy seems to be more questionable in second line setting. A retrospective/prospective trial conducted by GIM investigators showed mPFS 6.3 months, even though about 27% of patients had an ORR and almost 40% achieved durable disease control [8]. According to these data, the response rates published by Del Prete et al. showed a clinical benefit rate up to 50% with about 44% of the patients on T-DM1 for more than one year [7]. The mPFS was 10.5 months similar to the mPFS reported in EMILIA trial. On the other hand, other studies seem to suggest lower efficacy of T-DM1 following the triplet (TP and taxane). Findings from a real-word Italian study showed that patients with prior TP had significantly worse mPFS compared to those with prior trastuzumab only (5 versus 11 months, p value 0.01) [9]. The results from the phase II PERNETTA study, which prospectively evaluated the sequence of pertuzumab and trastuzumab with or without chemotherapy, also confirmed the lower efficacy of T-DM1 following the double block [6].

Regarding the T-DM1 activity in any line after TP, real word data are more concordant and similar to those reported in both the EMILIA and TH3RESA trials (mPFS 6.2 months) [4]. The exploratory analyses conducted by Urruticoechea in TP pre-treated patients enrolled in the CLEOPATRA and PHEREXA trials showed a mPFS 7.1 and 4.2 months, respectively [14]. A study by Lupichuk et al. confirmed a mPFS of 5.5 months in the pertuzumab-exposed group [12]. Similar evidence in terms of mPFS was reported in another multicenter Italian retrospective trial [11]. Despite that, both studies reported worse survival outcomes in the TP pre-pretreated population compared to the pertuzumab naïve population. Hence what happens in the unselected population treated with PT therapy and receiving T-DM1 is still matter of debate. Unfortunately, there is no scientific interest in prospectively set up clinical studies aimed at formally evaluating the sequence of TP and taxane followed by T-DM1. What happens in the unselected population treated with PT in first line therapy and receiving T-DM1 is still matter of debate. Is always difficult to analyze data derived from real world studies, but they may sometimes constitute valuable sources of information that cannot be derived from randomized clinical trials. For that reason, we performed this meta-analysis with the aim of exploring the real efficacy of T-DM1 after a PT-containing regimen. The real world studies analyzed in this article, examined individually, generate the hypothesis of a reduced effect of T-DM1 after the dual block therapy mainly in second-line setting. Despite this, the results from our meta-analysis showed that we could not draw certain conclusions on the ineffectiveness of the T-DM1 in the PT pretreated patients [9, 11]. Regardless of T-DM1 treatment lines, in the TP pre-treated population, the efficacy rate of T-DM1 seems to be similar to that previously reported in the EMILIA trial. In the second line setting, results from our meta-analysis are less impressive, however. Available data are not mature enough to confirm 2^nd^ line T-DM1 efficacy in TP pre-treated populations.

Of course, the analysis suffered from limitations due to the inclusion of data from retrospective trials and in some cases from analysis of population sub-group. On the other hand, the robustness of the meta-analysis performed is supported by the low heterogeneity of the studies included.

## Conclusion

Although the data from retrospective real word studies are difficult to analyze due to their heterogeneity, they represent the most powerful source of information when randomized trials are not practicable. Although T-DM1 represents the standard treatment recognized in all the international guidelines, its efficacy in a TP pretreated setting is still unknown. Available data from real life studies justify the doubts about the activity of drugs in TP pre-treated patients. Results from the current meta-analysis show that the efficacy of T-DM1 in any line after TP double-block seems to be similar to that previously reported in the EMILIA pivotal trial. In the second line setting, available data are not mature enough to confirm T-DM1 efficacy in the TP pre-treated population.

To date, there are no established proofs of T-DM1 ineffectiveness in TP populations, even if its activity may be negatively influenced by previous HER2 double block. In fact, data on second line setting T-DM1 are not mature enough to conclude T-DM1 efficacy in TP pretreated patients. Despite this, the available evidence support the notion that TP pretreated patients should receive T-DM1 as indicated in the guidelines.

## Supplementary Information

**Additional file 1:** 

## Data Availability

All the data and materials are reported in the manuscript.

## Abbreviations

T: Trastuzumab
P: Pertuzumab
MBC: Metastatic breast cancer
PFS: Progression free survival
ORR: Objective response rate
